# Error correction and statistical analyses for intra-host comparisons of feline immunodeficiency virus diversity from high-throughput sequencing data

**DOI:** 10.1186/s12859-015-0607-z

**Published:** 2015-06-30

**Authors:** Yang Liu, Francesca Chiaromonte, Howard Ross, Raunaq Malhotra, Daniel Elleder, Mary Poss

**Affiliations:** 1 Department of Statistics, The Pennsylvania State University, University Park, PA 16802 USA; 2 Department of Biology, The Pennsylvania State University, University Park, PA 16802 USA; 3 Department of Veterinary and Biomedical Sciences, The Pennsylvania State University, University Park, PA 16802 USA; 4 The Huck Institutes of the Life Sciences, The Pennsylvania State University, University Park, PA 16802 USA; 5 Bioinformatics Institute, School of Biological Sciences, University of Auckland, Auckland, 1142 New Zealand; 6 Department of Computer Science and Engineering, The Pennsylvania State University, University Park, PA 16802 USA; 7 Current address: Institute of Molecular Genetics, Academy of Sciences of the Czech Republic, Videnska 1083, Prague, 14000 Czech Republic

**Keywords:** Virus Population Dynamics, Next Generation Sequencing, FIV, Error Correction, Linear Mixed Model, Viral Coinfection

## Abstract

**Background:**

Infection with feline immunodeficiency virus (FIV) causes an immunosuppressive disease whose consequences are less severe if cats are co-infected with an attenuated FIV strain (PLV). We use virus diversity measurements, which reflect replication ability and the virus response to various conditions, to test whether diversity of virulent FIV in lymphoid tissues is altered in the presence of PLV. Our data consisted of the 3′ half of the FIV genome from three tissues of animals infected with FIV alone, or with FIV and PLV, sequenced by 454 technology.

**Results:**

Since rare variants dominate virus populations, we had to carefully distinguish sequence variation from errors due to experimental protocols and sequencing. We considered an exponential-normal convolution model used for background correction of microarray data, and modified it to formulate an error correction approach for minor allele frequencies derived from high-throughput sequencing. Similar to accounting for over-dispersion in counts, this accounts for error-inflated variability in frequencies – and quite effectively reproduces empirically observed distributions. After obtaining error-corrected minor allele frequencies, we applied ANalysis Of VAriance (ANOVA) based on a linear mixed model and found that conserved sites and transition frequencies in FIV genes differ among tissues of dual and single infected cats. Furthermore, analysis of minor allele frequencies at individual FIV genome sites revealed 242 sites significantly affected by infection status (dual vs. single) or infection status by tissue interaction. All together, our results demonstrated a decrease in FIV diversity in bone marrow in the presence of PLV. Importantly, these effects were weakened or undetectable when error correction was performed with other approaches (thresholding of minor allele frequencies; probabilistic clustering of reads). We also queried the data for cytidine deaminase activity on the viral genome, which causes an asymmetric increase in G to A substitutions, but found no evidence for this host defense strategy.

**Conclusions:**

Our error correction approach for minor allele frequencies (more sensitive and computationally efficient than other algorithms) and our statistical treatment of variation (ANOVA) were critical for effective use of high-throughput sequencing data in understanding viral diversity. We found that co-infection with PLV shifts FIV diversity from bone marrow to lymph node and spleen.

**Electronic supplementary material:**

The online version of this article (doi:10.1186/s12859-015-0607-z) contains supplementary material, which is available to authorized users.

## Background

The dynamics of lentiviral infection within a host have been intensively studied because they reveal important temporal and spatial features of virus-host interaction [1–3]. These interesting dynamics arise largely due to the unique lentiviral life history strategy that leaves a DNA copy (a provirus) of the viral RNA in the genome of an infected cell. The mutational spectrum attributed to a lentivirus population arises from errors introduced during the conversion of the RNA genome to DNA. Viral population structure is evident across tissues of a lentivirus-infected host in part due to the error rate in reverse transcription of the viral genome and in part due to the targeted movement of infected cells to specific tissues [1, 4]. The primary cells for lentiviral infection include monocytes and T cells, which migrate between sites of inflammation in tissue and lymphoid organs via the blood. Differential movement and subsequent activation of infected cells determine the diversity of viruses among host tissues. If an infected cell is activated at a site of inflammation, new progeny lentiviruses can be produced and infect naïve cells recruited to the site. Newly infected cells in a tissue will contain a provirus differing at several sites from the parental virus and indicate that virus replication has occurred. In contrast, expansion of an infected cell without production of virus and cell reinfection will increase the provirus census size without virus replication or increase in virus diversity. Thus virus diversity in a tissue will change depending on the number of infected cells migrating into or out of a tissue and virus replication in the tissue even if there is no change in census size.

Changes in lentivirus population dynamics under different conditions inform mechanisms that contribute to infection outcome. For example, cats experience insidious immune system dysfunction when infected with the lentivirus feline immunodeficiency virus (FIVfca; FIV hereafter) [5, 6], which presents a similar clinical profile as human immunodeficiency virus infected humans [6–8]. Cats infected with FIV derived from cougars (FIVpco; strain PLV) do not develop disease [9] but are protected against the loss of CD4 T cells, which is an indicator of FIV-induced immune dysfunction [10]. Data indicate that innate immunity may play a key role in PLV protection [9], and that humoral immune parameters do not significantly differ between cats infected with both FIV and PLV and those infected with FIV alone [11, 12]. We also observed that the dynamics of FIV-infected cells in the blood are significantly different in dual and single infection [13]. Cats infected with both FIV and PLV have a lower effective population size of FIV and the FIV population undergoes a bottleneck at 3–5 weeks post infection that further reduces the effective population size. In the present study, we investigate mechanisms contributing to the differential outcomes of FIV infection in the presence and absence of PLV by testing the hypothesis that FIV diversity in immune tissues is altered in the presence of PLV.

Next generation sequencing (NGS) approaches have recently been applied to study diversity in viral populations. These studies have identified low frequency substitutions (e.g. those that may be associated with drug resistance [14–16]) and changes in viral population diversity within hosts during infection [17–20] utilizing innovative methods to reconstruct individual viral haplotypes from NGS sequences [21, 22].

Nevertheless, evaluating diversity in a viral population from NGS data remains challenging because of the short size of the reads and the presence of errors whose rates are higher than in Sanger sequencing [23] and vary with the specific sequencing platform. The development of algorithms to differentiate between errors and actual genetic variants and/or to perform error correction in NGS data is an active area of research. These algorithms commonly utilize error thresholds [24] or Poisson/Binomial error distributions [18, 19, 25, 26] which can be site-specific. Thresholds or error distribution parameters are fixed in a variety of ways; e.g. using values from existing literature [24], values derived from quality scores [19, 26], or values estimated by computing errors from sequences of cloned samples obtained under conditions matching those of the samples under consideration [18, 25].

Importantly, these algorithms are used to detect “true” single nucleotide variants in the presence of error, rather than to reduce the contribution of errors to the observed signals of interest. In our analyses, these signals are *minor allele frequencies*; that is, the frequencies with which nucleotides different from the reference occur at any given position in the viral genome – so the problem can be rephrased by saying that, in addition to be able to separate “truly” non-0 frequencies from those that are non-0 only due to error, one would want to clean the former from error that they, too, contain. Unless observed signals are properly corrected, they will reflect a combination of errors and “true” biological signals – with an inflated variability that can influence subsequent statistical analyses. When the signals of interest are read counts (e.g. from RNA-Seq or ChIP-Seq), this inflation results in so-called over-dispersion [27–29]; since a Poisson distribution, where the variance is bound to equal the mean, is inadequate to model these counts, researchers often switch to a Negative Binomial distribution, where the variance can be larger than the mean [29]. Of course the issue of how to model simultaneously “true” biological signals and errors superimposed to them concerns also other quantities derived from high-throughput sequencing data; e.g., the minor allele frequencies we are interested in. Our error correction approach is based exactly on developing such a model.

We note here the existence of another broadly used class of algorithms that specifically target error correction working at the level of the reads. These utilize probabilistic clustering of reads within overlapping sequence windows; reads are aligned to the reference and error correction is accomplished by converting each aligned read to the consensus or the cluster centroid in the window [17, 21, 30], or removing rare reads [31]. While these algorithms have been shown to reduce per-base error, they are computationally expensive and work most effectively when sequencing error rates are substantially lower than substitution rates underlying the data [32].

In this study we use data generated by Roche 454 sequencing of partial viral genomes to investigate whether the presence of PLV changes the FIV population diversity in several lymphoid tissues of the cat host. Virus populations evolving over short infection times likely comprise a large number of very low-frequency minor alleles – with the ranges of “true” signals and errors substantially overlapping. As thresholding does not really accomplish error correction, and algorithms such as [17, 21, 30, 31] may not be effective in these settings, it was paramount for us to develop an alternative error correction approach.

To do so, we borrowed an idea used in background correction of microarray data. For such data, one can simultaneously model “true” signals and errors with an exponential-normal convolution, and perform error correction (i.e. reduce the error portion of the observed signals) using conditional expectations [33, 34]. Minor allele frequencies are continuous quantities, but their empirically observed distributions (especially when very low frequencies are abundant) are not necessarily well represented by an exponential-normal convolution. We therefore adapted the original idea modifying the model as to match empirically observed distributions.

After correcting minor allele frequencies with our approach, we analyzed them with an ANOVA framework and found significant evidence for tissue differences in FIV population diversity in the presence and absence of PLV. Importantly, when the data was processed by thresholding minor allele frequencies or running ShoRAH (an error correction algorithms based on probabilistic clustering of reads) [21, 30] this evidence was reduced or lost.

## Results

### Error correction

In this study we use NGS data to characterize FIV genetic diversity in tissues of infected cats in the presence or absence of PLV. Genetic diversity reflects the selective pressures, migration, and growth experienced by a virus population. Virus populations replicating under weak selection pressure will have an abundance of rare variants, which can carry key information about evolutionary processes. In this situation, removing minor allele frequencies below a threshold to eliminate errors may in fact remove relevant signal from the data -- and also more sophisticated error correction algorithms (e.g., based on probabilistic clustering of reads) may not be effective. Thus, before analyzing the data with an ANOVA framework, we developed and implemented our own error correction approach.

All sequencing reads from each library were aligned to a reference genome representing the cloned virus used in the infection experiments. Deletions and insertions in sequence tags were not considered, so for each nucleotide position along the genome there were 4 possible alleles (A, C, G, T) – one representing the reference, and 3 representing minor alleles. We computed frequencies of each minor allele at each position from each of 12 libraries (two libraries were available per ANOVA “treatment”; i.e. tissue and infection status combination).

To perform error correction, we started by considering an exponential-normal convolution model, as in the Robust Multi-Array Average (RMA) software [35–37]. RMA was developed for microarray data, which consists of continuous values of fluorescent intensities, and cannot be applied to discrete read counts data – e.g. from RNA-seq. However, with appropriate modifications, the RMA approach is suitable for our study because minor allele frequencies, albeit derived from read counts produced by DNA-Seq, are continuous quantities. Compared to thresholding, which only removes very low observed frequencies, a model that convolutes “true” signal and error allows us to account for the increased variability due to the latter, and to correct observed minor allele frequencies of any size by calculating conditional expectations – as is done in RMA (see Methods). Moreover, we can operate directly on minor allele frequencies instead of going back to reads and attempt to modify or remove erroneous ones – as in error correction algorithms based on probabilistic clustering. As we show below, utilizing a convolution model for the frequencies is computationally much faster, and in fact more effective for our type of data.

**Fig. 1:**
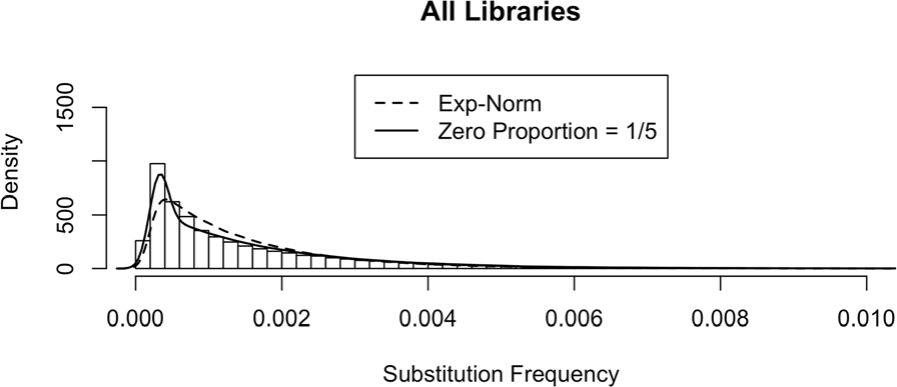
Histogram and simulated distributions: combined data. Minor allele (i.e. substitution) frequencies from all 12 libraries were pooled into the empirical distribution represented by the histogram. The dashed curve represents the distribution simulated from an exponential-normal convolution model with parameter values estimated on the data (Table 1). The solid curve represents the distribution simulated from the same model with parameters estimated on library pair 1 and 2 (Table 1) appropriately “spiked” with low frequency substitutions (see inset) to account for their abundance in high coverage sequencing data

**Fig. 2:**
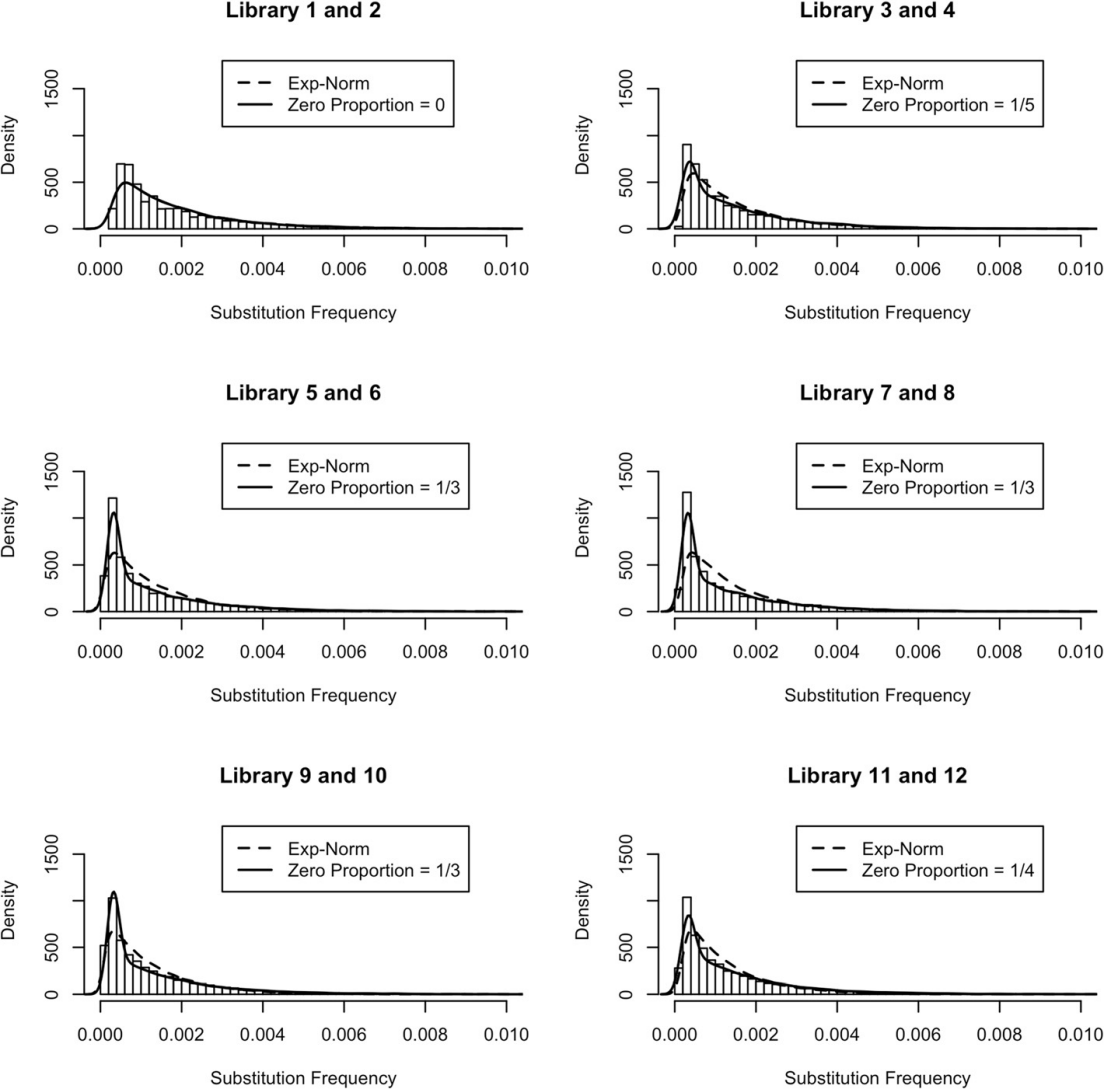
Histograms and simulated distributions; library pairs. Histograms represent empirical distributions for minor allele (i.e. substitution) frequencies for 6 library pairs, which are derived by pooling minor allele frequencies of the two replicates for each of 6 “treatment” conditions (tissue by infection status) represented by the 12 libraries. Dashed curves represent distributions simulated from exponential-normal convolution models with parameter values estimated for each library pair (Table 1). Solid curves represent distributions simulated from the same models with parameters estimated on the library pair 1 and 2 (Table 1) appropriately “spiked” with low frequency substitutions (see insets) to account for their abundance in high coverage sequencing data. Note that for the pair 1 and 2, where coverage is lowest, dashed and solid curves are undistinguishable. The differences between the two curves are largest for pairs with highest coverage (see Table 1)

If we model minor allele frequencies as an exponential-normal convolution (i.e. true variant frequencies drawn from an exponential distribution with independent normal errors additively superimposed), we can use the RMA software as is to estimate model parameters (rate α for the “true” exponential signal; mean μ and standard deviation σ for the normal error) and thus the underlying true variant frequencies as conditional expectations. The dashed curves in Figs. 1 and 2 represent distributions of minor allele frequencies simulated from exponential-normal convolution models with parameter values estimated using the original implementation of RMA (Table 1) on data obtained pooling all libraries (Fig. 1), and then pairs of libraries corresponding to the same tissue and infection status combination (Fig. 2; six pairs). It is apparent that, with the exception of the library 1 and 2 pool, the simulated distributions do not match the observed minor allele frequencies (histograms) at frequency ranges below 0.002. The mismatch is due to an excess of very low minor allele frequencies that is not captured by an exponential-normal convolution model. Interestingly, the data from library pair 1 and 2, which the model accurately captures, has the lowest sequencing coverage. This suggests that higher sequencing coverage increases the number of low frequency substitutions, resulting in extra sequencing error that cannot be accounted for within the exponential-normal modeling framework. The failure of the exponential-normal convolution model to capture minor allele frequencies in high coverage libraries can result in substantial biases in the RMA parameter estimates for these libraries (this may account for some of the differences in parameter estimates seen in Table 1) and requires that the model be modified.

**Table 1:** Parameter Estimates for the Exponential-Normal Convolution Model

Library pairs	${\widehat{\alpha}}^a$	${\widehat{\mu}}^b$	${\widehat{\sigma}}^c$	Total Reads	Average Coverage
1 & 2	637	3.2×10^-4^	5.1×10^-5^	63439	1810
3 & 4	750	2.4×10^-4^	4.7×10^-5^	90839	2775
5 & 6	810	1.4×10^-4^	1.7×10^-5^	133801	4236
7 & 8	813	1.8×10^-4^	2.1×10^-5^	132589	4078
9 & 10	865	1.4×10^-4^	3.6×10^-5^	177873	5530
11 & 12	888	2.1×10^-4^	6.6×10^-5^	118396	3916
All	777	1.6×10^-4^	5.1×10^-5^	716937	3724

^a^ $\widehat{\alpha}$: estimated rate parameter for the exponentially distributed signal (obtained anchoring the 75^th^ percentile of an exponential distribution to the 75^th^ percentile of all frequencies minus the estimate of μ (i.e. the overall mode); ^b^ $\widehat{\mu}$: estimated mean for the normally distributed error (fixed at the overall mode); ^c^ $\widehat{\sigma}$: estimated standard deviation for the normally distributed error (obtained “doubling” the spread on the left of the overall mode)

To better capture the peak at very low minor allele frequencies, we introduced a point mass at 0 with proportion p as part of the modeling of the “true” minor allele frequencies. Our modified model is thus represented by a mixture of a point mass at 0 (weight p) and an exponential (weight (1-p)) providing the signal, which is then convoluted with a normal noise. Note that there are now four parameters to estimate in the modified model: the proportion p, the exponential rate α, and the normal mean μ and standard deviation σ. We did not attempt their joint estimation on all library pairs; instead, we proceeded as follows. We considered the estimates of α, μ and σ obtained with the original RMA on the library pair 1 and 2; these were $\widehat{\alpha}$ = 637, $\widehat{\mu}$ = 3.2 (10^−4^) and $\widehat{\sigma}$ = 5.1 (10^−5^). Because this pair had a good fit to the exponential-normal convolution model, and because all libraries share the same preparation protocols and sequencing platform, we fixed those values for all other library pairs, and then estimated p separately for each pair using a grid search between 0 and 1 to find a satisfactory match between empirical and simulated distributions (see inserts in Fig. 2; the same was done for the pool of all libraries – insert in Fig. 1). The very good match between histograms and distributions simulated from the modified model with parameter values selected as described above (solid curves in Figs. 1 and 2) suggests that our approach works well: an exponential-normal convolution model with parameters α, μ and σ estimated from the library pair 1 and 2, when appropriately “spiked” at 0 with a proportion p specific to each library pair, does provide a good reconstruction of the empirical distributions for all library pairs.

Given the estimated model parameters, we corrected minor allele frequencies in two stages. First, we removed the lowest minor allele frequencies, which are the ones most likely due to error alone. The minimum frequency observed in library pair 1 and 2 was 0.024 %; we kept all frequencies in libraries 1 and 2 and removed from other libraries all frequencies ≤ 0.023 %. From another perspective, this corresponds to discarding the 0.1 % of minor alleles with lowest frequencies under the exponential-normal convolution model with parameters estimated on libraries 1 and 2 – i.e. the pair that is consistent with such a model. Table 2 shows minimum frequency and number of minor alleles before and after removal for each of the 12 libraries. As can been seen from the table, the library pairs with lower minimum frequency and a larger number of removed minor alleles do indeed correspond to those with larger estimates of the 0-“spiking” parameter (p), which further confirms that excesses in very low minor allele frequencies must be accounted for when processing the data. Note that each of the removed minor allele frequencies is associated with a sequence position along the virus genome; in practice, removing a frequency means “reassigning” it to the reference nucleotide at that position (see Methods).

**Table 2:** Minor Alleles Before and After Removal

Library	No. minor alleles	Minimum frequency	No. minor alleles
	(before correction)	(before correction)	(after correction)
1	6253	2.4×10^-4^	6253
2	6526	2.6×10^-4^	6526
3	7911	1.8×10^-4^	7666
4	7083	1.9×10^-4^	6919
5	7314	1.7×10^-4^	7092
6	8588	1.1×10^-4^	6887
7	7839	1.7×10^-4^	7280
8	8344	1.4×10^-4^	7390
9	7581	1.7×10^-4^	7469
10	10965	0.7×10^-4^	8915
11	7711	1.5×10^-4^	7224
12	8744	1.1×10^-4^	7964

In the second error correction stage, we adjusted all remaining minor allele frequencies using the exponential-normal convolution model with parameters $\widehat{\alpha}$ = 637, $\widehat{\mu}$ = 3.2×10^−4^ and $\widehat{\sigma}$ = 5.1×10^−5^. Specifically, we replaced each observed frequency with the conditional expectation of the signal given the frequency from the convolution model (see Methods). In summary, our error correction procedure first removes minor alleles frequencies likely to be due to error alone, and then adjusts the remaining minor allele frequencies as to reduce the error component they carry. The resulting error-corrected minor allele frequencies were used in the subsequent ANOVA analyses.

### FIV genetic diversity across tissues and single or dual infection status

Our previous results demonstrated that FIV effective population size in peripheral blood cells is lower in the presence of PLV [13]. Because T cells in the blood are in transit between tissues and lymphoid organs, we hypothesized that the decrease in FIV effective population size in the blood was due to an affect of PLV on FIV tissue replication and migration. Since each cat in the study provided multiple observations (from the three sampled tissues) we adopted a split-plot ANOVA scheme, which comprised the fixed effects of tissue and infection status (single or dual), their interaction, and a random effect for cats nested within infection status (see Methods). Here we concentrate on infection-related effects, i.e. infection status fixed effects and tissue by infection status interaction effects, as a direct test of the hypothesis. The results for tissue fixed effects are provided in Figure S1 (see Additional file 1). Among several genetic diversity measures computed from the error corrected minor allele frequencies, which could be used as response variables in our analysis, we considered total number of conserved positions, frequency of transitions and frequency of transversions for the complete 3′ genome, and separately for each of the four constituent genes. We also considered the substitution frequencies at each of the 4603 nucleotide positions in the 3′ portion of the FIV genome under evaluation. Each response was appropriately transformed to be amenable for ANOVA analysis (see Methods).

**Fig. 3:**
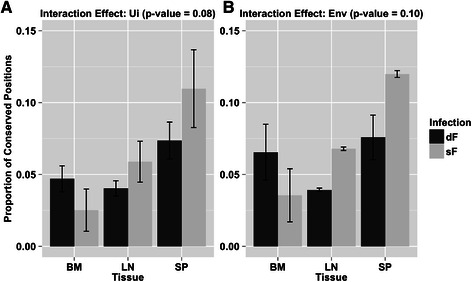
Significant tissue by infection status interactions for the number of conserved positions in the FIV UI (**b**) and *Env* (**b**) genes. Tissues are indicated as BM (bone marrow), LN (lymph node), and SP (spleen); infection status is indicated by sF (single infection with FIV) and dF (dual infection with FIV and PLV). Because the length of UI (the dUTPase and integrase portion of *pol*) and *env* are different, the vertical axis represents the proportion of conserved positions (number of conserved positions divided by gene length). The data was log transformed for ANOVA

In tissues with active viral replication or immigration of infected cells, viral genetic diversity will be high and the number of positions in the viral genome without any substitutions (conserved positions) will be low. The ANOVA results for conserved positions indicated that the dUTPase and integrase portion of FIV *pol* (UI) and envelope (*env*) genes have significant interaction effects of tissue and infection status (p-values ≤ 0.1, Fig. 3). The number of conserved positions in FIV UI and *env* sequences was higher in bone marrow of dual compared to single FIV infected cats. In contrast, FIV derived from lymph node and spleen of dual infected cats had fewer conserved sites than did FIV from those tissues in single infected cats. These results suggest that, based on overall viral sequence diversity, bone marrow is a preferred site of FIV replication or immigration of virus-infected cells. However, in the presence of PLV, FIV replication and trafficking of infected cells are displaced from bone marrow and are more prominent in mesenteric lymph node and to a lesser extent in spleen.

**Fig. 4:**
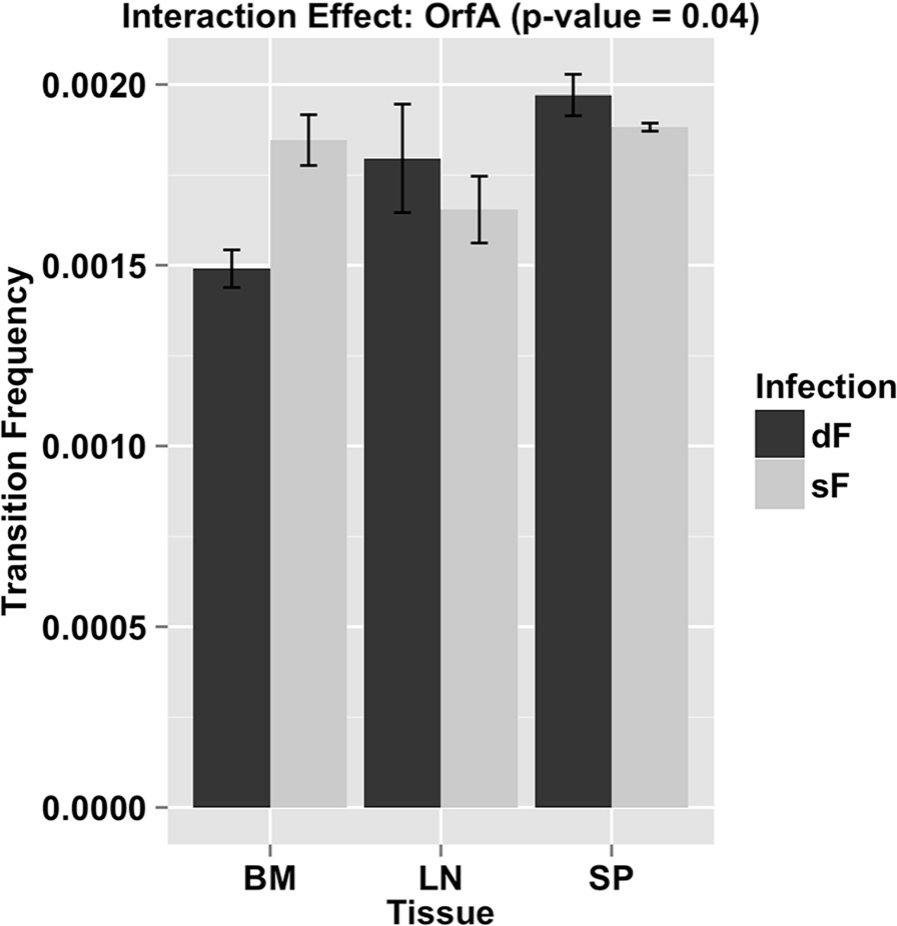
Significant tissue by infection status interaction for the frequency of transitions in the FIV *Orfa* gene. Tissues are indicated as BM (bone marrow), LN (lymph node), and SP (spleen); infection status is indicated by sF (single infection with FIV) and dF (dual infection with FIV and PLV). The vertical axis represents transition frequency. The data was logit transformed for ANOVA

In addition to overall viral genetic diversity, the types of substitutions that occur are also informative to virus dynamics. Transitions are the most common substitution in the genome of replicating retroviruses. The FIV *orfA* gene showed a significant interaction effect for transitions (p-value < 0.05; Fig. 4). The transition frequencies in FIV *orfA* sequences were lower in bone marrow and elevated in lymph node and spleen in the presence of PLV. There were no significant infection-related effects associated with the frequency of transversions. These results are consistent with the findings in Fig. 3 showing that PLV infection affects FIV diversity in bone marrow.

**Fig. 5:**
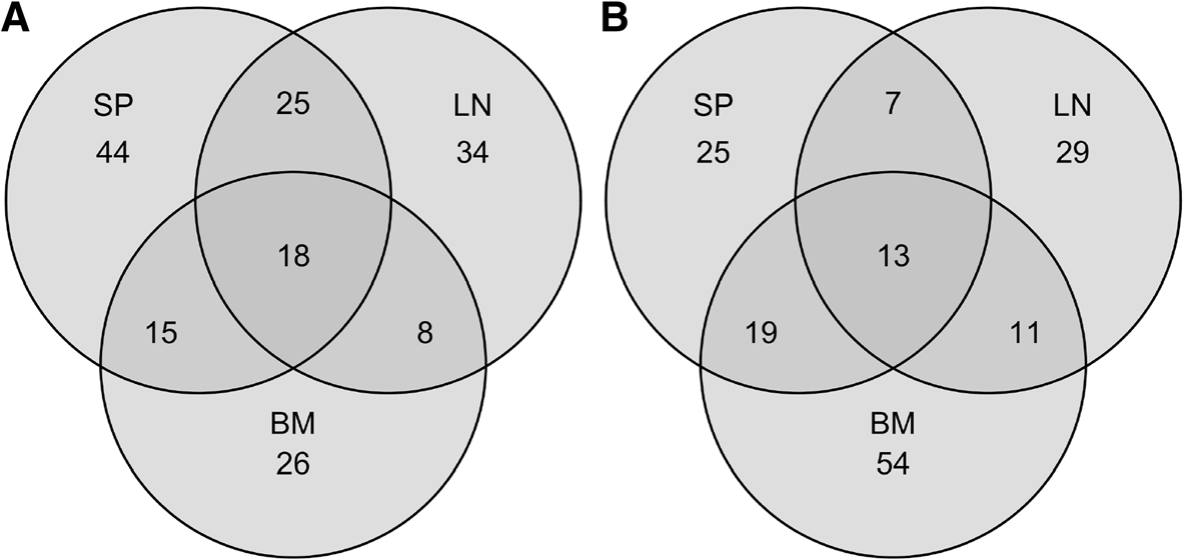
Tissue distribution of substitutions with significant infection-related effect. Substitutions with significant infection-related effects (242) are grouped based on whether they have higher frequencies in dual FIV-PLV infections (**a**) or in single FIV infections (**b**). For each group, the panel shows allocation of these substitutions to tissues indicated as BM (bone marrow), LN (lymph node), and SP (spleen)

Although substitutions detected in NGS data cannot be definitively assigned to individual viral genotypes, deep sequencing does provide a comprehensive assessment of substitution frequencies for the entire viral population at each position in the genome. We ran ANOVAs for logit-transformed substitution frequencies for each of 3 minor alleles at each of 4603 genomic positions. 242 substitutions with significant infection-related effects were identified after adjusting p-values for multiple testing (see Methods); 13 carried infection status effects and 229 had tissue by infection status interactions (see Additional file 2). In contrast to analyses at the genome or gene level (see above), most of the 242 substitutions were transversions (Table 3). This is likely because features at the genome or gene level arise as composites of all individual substitutions comprised in a large genomic interval. The substitution sites with significant infection-related effects were distributed across FIV genes as follows; 52 were found in UI (covering 4.9 % of UI positions), 38 in *vif* (5.0 % of *vif* positions), 9 in *orfA* (3.7 % of *orfA* positions) and 143 in *env* (5.6 % of *env* positions). Moreover, the majority of positions with higher substitution frequency in the FIV genome demonstrating significant infection-related effects were from spleen in the presence of PLV (Fig. 5a) but from the bone marrow in the absence of PLV (Fig. 5b). Thus our data based on substitution frequencies at individual sites are consistent with those on the number of conserved sites and transition frequency in genes and support that an important effect of PLV on FIV is a shift of FIV replication away from bone marrow.

**Table 3:** Substitutions Matrix Of Minor Alleles With Frequencies Significantly Affected By Infection-Related Effects

To	A	C	G	T
From				
A	-	37	23	27
C	14	-	14	10
G	11	24	-	20
T	26	7	29	-

### Performance of our error correction approach

To evaluate our error correction approach we considered its sensitivity in terms of number of retained minor alleles and, relatedly, the statistical power it affords in detecting effects on FIV diversity through ANOVA. We processed the data with simple thresholding and repeated our ANOVA analyses. If we remove minor allele frequencies using a threshold of ≤ 1 %, which is commonly done [24], we lose most of the minor alleles in our data and practically all significant effects in ANOVA . If we use a threshold of ≤ 0.05 %, the reported 454-platform substitution error rate [38], we retain more alleles and some significant effects, but still lose power to detect interactions at gene and genome scales. If we use a threshold of 0.023 % (i.e. the cut-off used in the first stage of our correction procedure, but without implementing the second stage where minor allele frequencies above threshold are corrected) we retain significant effects at gene and genome scales but with elevated p-values (weakened significance) compared to the full implementation of our approach (full comparisons in terms of number of detected minor alleles and ANOVA p-values are provided in Tables S1 and S2, see Additional file 3). Moreover, without correcting frequencies above threshold, we would likely increase false positives in the individual site analysis by unduly increasing frequency differences at sites where some minor alleles fall below threshold in some libraries and above in some others. These arguments suggest that even selecting a threshold based on model considerations and the data at hand is not enough; an effective error correction approach must also account for the error component carried by minor allele frequencies that survive the cut-off.

We also compared our approach to ShoRAH, which performs error correction using probabilistic clustering of reads [21, 30]. Similar to 1 % thresholding, ShoRAH retained very few minor alleles (Table S1, see Additional file 3) and led to lower power in detecting interaction and tissue effects at gene and genome scales – with only one tissue effect identified as significant (Table S2, see Additional file 3). Importantly, we also compared computational burden (running time of the correction steps; Table S3, see Additional file 3). Overall, our approach took less than a second per library, while ShoRAH took hours. This very large difference is due to the fact that ShoRAH performs expensive operations on a huge number of reads, while our approach perform inexpensive operations directly on the minor allele frequencies. Thus, for studies focusing on minor allele frequencies our approach, in addition to being more sensitive and affording higher power in subsequent statistical analyses, is computationally much more convenient.

### Enzymatic deamination and asymmetric substitution analyses

**Fig. 6:**
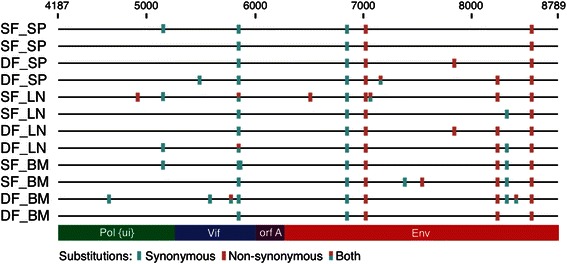
Regions of asymmetric substitutions. Each mark represents a position, or contiguous region, where the pattern of substitutions was significantly asymmetric (p-value ≈ 0). Tissues are indicated as BM (bone marrow), LN (lymph node), and SP (spleen); infections status is indicated as SF (single infection) and DF (dual infection). Reference sequence coordinates are showed on the x-axis

Cytidine deaminases can restrict retroviral replication by editing the viral genome during reverse transcription and are active in PLV infections in cat [39]. Although some viruses accumulate extensive G to A substitutions across the genome as the result of host enzymatic editing, enzymatic deamination is limited to target recognition sites in regions of the genome that are comprised of single stranded DNA [39]. We used two separate approaches to determine whether our sequence data contain evidence of enzymatic editing causing a substitution bias. First we performed a test specifically designed to detect asymmetry in the substitution matrix [40–42]. Given the duration of the infections, analyses of asymmetry in substitution provide an integrated summary of the effects of mutation, selection and random drift on the inoculated sequence. There were a total of seven sites with a significantly elevated asymmetry index in more than one library, but none involved the recognition triplet for cat cytidine deaminase activity GGA, or G to A substitutions (Fig. 6). Asymmetry was identified in some regions in all libraries; asymmetry at other multiply observed sites was not restricted to specific tissues or infection regimes.

As a second approach, we individually evaluated a cytidine deaminase recognition triplet GGA, which is present at 146 trinucleotide sites of the 3′ portion of the FIV genome. Using a Wilcoxon signed rank test we found that substitutions of the first G in the GGA triplet to A occur at significantly higher frequencies in the absence of PLV (p-value < 0.001), while substitutions to A at the second G in the GGA triplet do not show a significant difference between FIV sequences from single and dual infection.

## Discussion

The most pronounced effect of a concurrent PLV infection on FIV population dynamics in peripheral blood cell is a decrease in FIV effective population size and a transient population bottleneck within a month of FIV infection [13]. We hypothesized that changes observed previously in blood could reflect differences in tissue replication of FIV and/or migration patterns of FIV-infected cells when PLV is present. Our results provide evidence that the presence of PLV affects FIV population dynamics in tissues, with an apparent shift of replication or immigration of FIV-infected cells from bone marrow to both spleen and mesenteric lymph node.

High throughput sequencing approaches provide an opportunity to evaluate the full mutational spectrum in the viral population but analysis of short read data is challenging. Analysis of our experimental data was additionally difficult because cats were inoculated with a cloned virus. FIV evolutionary rates are estimated at 3 x 10^−3^ substitutions per site per year [43]. Because few of these substitutions become fixed in the population, we expected an abundance of low frequency variants in a population of replicating viruses. Thus, in order to be able to fully exploit the rich information in NGS data to address our hypothesis, we needed to properly account for error in our libraries, which would obscure the signal from low frequency substitutions.

Both our error correction approach and subsequent statistical treatment of the data advance analysis of viral diversity based on high throughput sequencing data. Instead of removing minor allele frequencies below a threshold, which we demonstrate can eliminate or weaken signal, we considered a convolution model that combines an exponential signal with normal error, and allows us to derive error-corrected variant frequencies as estimated conditional expectations. This idea was first implemented in the RMA software for application to microarray data [33, 34]. However, in order to effectively apply it to minor allele frequencies from our sequencing data, which has an abundance of very small frequencies, we had to modify the model incorporating an “extra amount” of 0-signal. We showed that this modification works quite effectively, in the sense that the modified model reproduces the observed frequency distributions, and that it is more critical the higher the coverage of a library – since higher coverage tends to increase the number of very small frequencies. We note that parameter estimation for the modified model is implemented in an “ad hoc” manner; a more rigorous estimation procedure is left for future development.

Analytically, using a model that convolutes “true” signal and error accounts for error-induced inflation of variability in the observed signal (see Methods). This is logically similar to accounting for over-dispersion (e.g. due to sequencing errors, sample preparation protocols, etc.) in state-of-the-art approaches for analyzing read counts from NGS [27–29]. In effect, an appropriate convolution model allows one to exploit information in very small frequencies when correcting larger ones.

Compared to simple thresholding of minor allele frequencies, our approach is more sensitive and affords increased statistical power in subsequent statistical analyses. Our approach also appears to guarantee better sensitivity and statistical power than ShoRAH – likely because our data is characterized by an abundance of very small minor allele frequencies, which approaches based on probabilistic clustering of reads tend to over-correct. In addition, our approach is orders of magnitude faster – because correcting at the level of the aligned reads can be very time-consuming, especially for libraries with high coverage and deep sequencing. In contrast, our approach corrects directly the minor allele frequencies requiring only a few, computationally fast estimation and correction steps.

In summary, our correction approach does manage to effectively exploit information in very abundant small minor allele frequencies – which is not exploited by, and in fact hinders, other recently proposed algorithms [17, 21, 30].

This makes it more sensitive and affords increased power to ascertain biological effects in subsequent statistical analyses. Our approach is also computationally much leaner, providing huge running time gains.

High rates of asymmetric substitution can be due to a host defense mechanism that entails enzymatic deamination of cytidine residues in the viral genome, resulting in an excess of G to A mutation [44]. Viral replication is disrupted through the production of premature termination codons in viral proteins or the creation of proteins with sub-functional folding properties. FIV can replicate in cells expressing cytidine deaminase because it encodes an accessory protein, *vif*, which protects the viral genome from cytidine deamination, in part by increasing enzyme degradation [45, 46]. The *vif* from PLV, which is derived from a cougar, does not protect the PLV genome from domestic cat cytidine deaminase. Because there is evidence for cytidine deamination of the PLV genome [39], we reasoned that elevated levels of cytidine deaminase might reduce FIV replication in some tissues in a co-infection with PLV. We queried our comprehensive viral sequence data set for evidence of asymmetry in general, and of an increase in the minor allele frequency of G to A substitutions at an enzyme trinucleotide target site, GGA. Our data do not support an increase in G to A substitution frequency in the FIV genome in the presence of PLV. In fact, a significant increase in G to A substitutions at the first G in the trinucleotide target GGA was detected in the FIV genome in single infections. While these data do support that processes acting on the FIV genome differ in the presence and absence of PLV, the lack of evidence for G to A substitution bias in the FIV genome in dual infection suggests that restriction by cytidine deaminase activity is not the primary mechanism for observed changes in tissue dynamics.

High throughput sequence analysis of retrovirus genomes affords a unique perspective on viral dynamics. Because retroviruses integrate their genome into the genome of the infected cell and the target cells for retroviral infection are migratory, an increase in the overall virus population diversity of a sampled tissue can be caused either by active virus replication in the tissue, with ensuing integration into susceptible cells, or by recruitment of migratory cells, which were infected at a different time or location. Our ANOVA results support a significant tissue by treatment interaction. The FIV population diversity is highest in bone marrow in the absence of PLV as evidenced by more genomic sites affected by substitutions in the dUTPase/Integrase portion of *pol* and *env* and by an increased frequency of transitions in *orfA*. In addition the substitution (minor allele) frequencies were higher in FIV derived from bone marrow of infected cats. In contrast, in the presence of PLV, FIV population diversity is decreased in bone marrow and elevated in spleen and lymph node, suggesting a shift in target tissue for virus replication. Collectively, these results support our hypothesis that a primary mechanism by which PLV attenuates virulent FIV infection is altering the within-host dynamics of infected cells and/or virus replication. By reducing immigration of infected cells and/or FIV replication in bone marrow, PLV could confer protection to hematopoietic cells essential to maintain immune system health. Our analyses, which rely on a thorough procedure to account for errors in NGS, provide an important advance in using high throughput approaches to interrogate tissue specific virus replication in different treatment regimes.

## Conclusions

This article demonstrates a systematic framework to access the full frequency spectrum of genomic diversity in viral populations based on high-throughput sequencing of viral genomes. We address the problem of separating signals from the errors intrinsic in NGS technologies, which is critical to understand the underlying biological phenomena. The issue is particularly important when it is necessary to use information from low frequency variants. We propose an error correction approach that is easy to implement, computationally fast, and provides good performance in distinguishing rare variant signals within data characterized by an abundance of very small minor allele frequencies. As a consequence, the approach also guarantees good statistical power when using ANOVA based on linear mixed models on the error-corrected data. Doing so, we find evidence that FIV population dynamics change among tissues in the presence of PLV.

## Methods

### Viral sequence data

The experiment comprised 4 cats infected with FIV alone (single infection) and 4 cats infected with FIVpco isolate PLV one month prior to FIV inoculation (dual infection) [11]. Viral sequences were obtained from tissue samples taken at 150 days post infection as previously described [13]. Briefly, the 3′ portion spanning 4187–8789 bp of the FIVC36 genome [AY600517] (Figure S2, see Additional file 4) was amplified from DNA from 3 tissues (bone marrow, mesenteric lymph node and spleen) of each cat. Proviral copy number in each tissue was determined by quantitative PCR based on three viral genes (Table S4, see Additional file 3). Five to ten PCR were generated for each sample to assure adequate sampling of viral diversity. Each PCR had a template input of 10–50 copies of the FIV genome, which we have previously showed is sufficient template to produce a visible band for downstream analysis. PCRs were purified using Qiagen PCR purification columns and quantified. Equal concentrations of the PCRs obtained from replicate sampling of each cat’s tissue were pooled. Of the 24 samples (eight cats, three tissues per cat), those from two cats of the same infection status and tissue were pooled into one sequencing library due to cost considerations. The resulting 12 libraries (two for each of the six combination of infection status – single or dual, and tissue – bone marrow, lymph node and spleen) were generated by nebulization and adapter ligation, and sequenced using the 454 platform (Table 4). The sequence data thus produced had quality scores for downstream processing, but was *not* error corrected (454 does not implement an error correction).

**Table 4:** Library Information

Library	Cat number	Infection	Tissue
1	97/99	Single	Spleen
2	02/06	Single	Spleen
3	89/93	Dual	Spleen
4	03/05	Dual	Spleen
5	97/99	Single	Lymph node
6	02/06	Single	Lymph node
7	89/93	Dual	Lymph node
8	03/05	Dual	Lymph node
9	97/99	Single	Bone marrow
10	02/06	Single	Bone marrow
11	89/93	Dual	Bone marrow
12	03/05	Dual	Bone marrow

### Sequence pre-processing

The average length of raw reads was 299 bp. We applied a strict read quality filter using a threshold of 0.02 in CLC Genomics Workbench (version 5.1) [47], which resulted in high-quality reads with average length of 263 bp. Using the same software, these were mapped to the complete FIVC36 genome (9466 bp), which is the sequence of the cloned virus used to infect cats in this study. Mapping parameters were implemented as follows: Insertion Cost = 3; Deletion Cost = 1; Mismatch Cost = 2; Length Fraction = 0.9; Similarity = 0.9; Global Alignment; Ignoring Non-specific Match. After alignment, all insertions in reads leading to a gap in the reference sequence were removed to maintain the reference at 9466 bp, and the corresponding 4603 bp sequence from the 3′ half of FIV genome was utilized for subsequent analyses.

### Error correction using a convolution model

The dataset corresponding to each of the 12 libraries consists of rows, representing the read coverage, and columns, representing sequence variants in the reads, for every position in the 3′ portion of the FIVC36 genome. The frequency of each variant (minor allele) at every position in each library is regarded as a combination of a “true” biological signal and error; our aim is to correct for the latter.

The Robust Multichip Average (RMA) software in Bioconductor [48] proposes a background correction procedure for genome-wide microarray data based on an exponential-normal convolution model. For the purpose of parameter estimation, RMA treats the frequencies at or below the overall frequency mode as the “left half” of a normal error distribution with mean μ and standard deviation σ. μ is thus estimated by the overall mode itself, and σ by “doubling” the spread on its left (see [34]). We actually truncated the normal at 0 to better reflect the absence of negative frequencies when estimating the standard deviation. Frequencies above the overall mode are treated as reflecting, by and large, an exponential “true” signal distribution with rate α – shifted to the right by μ (Figure S3, see Additional file 5). Following the RMA-75 implementation [34], we took the 75^th^ percentile of all frequencies minus the estimated μ as the 75 % percentile to anchor an exponential distribution, and estimated α through its cumulative distribution function. Simulation studies show that this approach guarantees a conservative and robust estimation of the signal rate [34].

For the purpose of error correction, we first remove from each library all minor allele frequencies below the 0.1 % percentile of the exponential-normal convolution model estimated on the library pair 1 and 2 (this corresponds to a threshold of 0.023 %). The removed frequencies are “reassigned” to the reference nucleotide at their sequence positions. Second, we adjust the remaining minor allele frequencies using the exponential-normal convolution model estimated on the library pair 1 and 2. Each observed frequency is replaced with the conditional expectation of the signal given the observed frequency itself, as illustrated in the scheme below. All calculations are implemented in the statistical computing environment R, version 2.15 [49].

### Scheme for error correction

- Set up the exponential-normal convolution model X = S + E, where X is the (observable) variant frequency, S the “true” signal ~ Exponential (α), and E the error ~ Normal (μ, σ^2^) independent of S.
- The expected value of the signal given the frequency is:

$$ E\left(S\Big|X=x\right)=a+b\left(\frac{\phi \left(\frac{a}{b}\right)-\phi \left(\frac{x-a}{b}\right)}{\phi \left(\frac{a}{b}\right)-\phi \left(\frac{x-a}{b}\right)-1}\right), $$

$$ with\ a=x-\mu -{\sigma}^2\alpha,\ and\ b = \sigma $$

and can be estimated using the parameter estimates $\widehat{\mu},\ \widehat{\sigma},\ \widehat{\alpha}$.

- To compute the error-corrected frequency, fix a quantile *q* of the estimated Exponential-Normal convolution distribution (we used *q* = 0.1 %), and set:

$$ {x}_{corrected}=\Big\{\kern1em \begin{array}{c}0\kern1em \\ {}\kern1em \widehat{E}\left(S\Big|X=x\right)\kern1em \end{array}\begin{array}{cc}\kern1em ,\kern1em & \kern1em  if\kern0.5em x\kern0.5em <q\kern1em \\ {}\kern1em ,\kern1em & \kern1em  otherwise\kern1em \end{array} $$

- For all cases with *x*_*corrected*_ = 0, attribute the frequency *x* back to the reference allele.

### Accounting for error variability in a convolution model

The “true” exponential signal has variance Var(S) = α^−2^. Convolution with the independent normal error inflates the variance of the observed signal to Var(X) = Var(S + E) = Var(S) + Var(E) = α^−2^ + σ^2^. The model thus accounts for error variability, and so does the correction based on it – where the observed signal is replaced with the expected value of the “true” signal given the observed signal itself.

### Error correction using ShoRAH

ShoRAH is a software to perform error correction, reconstruct viral haplotypes, and estimate their relative frequencies in a population. It takes as input a reference genome and a set of reads aligned to the reference. Error correction is performed with probabilistic clustering of reads in a moving window, using a Dirichlet Process Mixture Model (DPM) [21, 30]. Utilizing the same reads alignments from which we derived the minor allele frequencies then corrected with our approach, we ran the shorah.py script with default parameters (window size = 201 bp; *α* parameter for the DPM = 0.1) on each of our 12 libraries. We took error corrected reads from the intermediate output files marked by the suffix *“_cor.fas”*, re-aligned them to the reference using the CLC Genomics Workbench (see *Sequence Pre-processing* above), re-computed minor allele frequencies, and repeated the ANOVA analyses (see below) across the 12 libraries. We then compared results, in terms of number of minor alleles surviving correction in each library (Table S1, see Additional file 3), in terms of ANOVA p-values (Table S2, see Additional file 3), and in terms of running time of the correction steps (Table S3, see Additional file 3) to those obtained with our error correction approach.

### Analysis of variance

With the error-corrected minor allele frequencies, we use analysis of variance (ANOVA) based on a split-plot design linear mixed model to determine if infection status (whole plot factor; single and dual), tissue (the split plot factor; spleen, bone marrow and lymph nodes) or their interaction significantly affect virus genetic variation. We also introduce a random effect to account for differences induced by the 8 cats involved in the experiment. The model equation we employed is therefore of the form

$$ y = mean + infection + cat(infection) + tissue + infection* tissue + error $$

where *y* is a response (see below), *mean* is the overall mean; *infection, tissue* are main fixed effects; infection*tissue is the fixed interaction effect; *cat(infection)* is the cat random effect (nested in infection); *error* is the random error.

As responses (y) we take measurements reflecting virus replication in each environment (e.g. number of conserved positions, transition frequency, transversion frequency, individual substitution frequency). We consider these at the genome level, at the gene level, and at individual sites along the genome. We also transform them by natural logarithm (for counts) and logit (for frequencies) to satisfy the basic assumption underlying ANOVA (zero counts or frequencies are shifted right by a very small amount prior to transformation).

When running multiple tests (e.g. on ANOVA effects for single genomic positions) we employ the Benjamini Hochberg method for False Discovery Rate (FDR) control on the expected proportion of incorrectly rejected null hypotheses. All calculations are implemented in the statistical computing environments SAS, version 9.2 [50] and R, version 2.15 [49].

### Asymmetric substitutions analysis

This analysis identifies the cumulative effect of mutation, drift and selection that has occurred on FIV as it evolves from the sequence used to initiate infection. Rates of substitution were estimated from a nucleotide association matrix in which the columns represent the nucleotides occurring in the infecting (reference) strain and the rows represent the nucleotides occurring among the aligned reads. The cells contained the frequency of the associations computed along a sliding window of the alignment and centered on each site. The variable-width window was symmetrical about the site with a width the minimum sufficient to include all four nucleotide bases in the reference sequence. The cell frequencies in each column (reference base) were scaled by dividing by the number of times that base occurred in the window in the reference sequence.

Asymmetry in substitution was characterized by an index, *AI*, obtained by summing the differences in off-diagonal elements and dividing by the sum of all cells, using the equation

$$ AI = \frac{{\displaystyle {\sum}_{i\ne j}}\left|{x}_{ij}-{x}_{ji}\right|}{{\displaystyle {\sum}_i}{\displaystyle {\sum}_j}{x}_{ij}} $$

where *x*_*ij*_ is the number of occurrences where base *i* in the reads was associated with base *j* in the reference sequence.

The nucleotide association table for each window was tested for asymmetry using an R-language script provided by Ababneh et al. [42]. This script computed the overall asymmetry [40] and partitioned it into a component due to the marginal distributions, corresponding to the test of [41], and a component due to internal asymmetry. Sites where the associated *P*-value for overall asymmetry approached zero were noted. These sites all have associated high values of the asymmetry index AI (Figure S4, see Additional file 6). While the values of AI are not independent due to the sliding window used to compute them, they offer a separate measure of asymmetry.

### Wilcoxon signed rank test

Wilcoxon signed rank test, a non-parametric statistical hypothesis test, was used to compare the G to A substitution frequencies of 146 occurrences of the GGA trinucleotide between single and dual infections. The substitution frequencies were averaged over libraries corresponding to the same infection status. We performed comparisons for the first and second G separately. Wilcoxon signed rank test is used to test the null hypothesis that the median difference of G to A substitution frequencies between single and dual infections is zero.

### Availability of supporting data

The data sets supporting the results of this article are included within the article (and its additional file). The original sequence data sets are available in Dryad [submission in progress].

## Additional files

Additional file 1: Figure S1.
**Significant Tissue Main Effects For The Frequency Of Transversion And The Number Of Conserved Positions.** Results of ANOVAs run for frequency of transversions (after logit transformations) in the FIV UI (A), and *env* (B) genes, and for the whole genome (C). The vertical axis represents transversion frequency. The dUTPase and integrase portion of the FIV *pol* (UI), envelope (*env*) genes, and the whole genome showed significant tissue main effects (p-values < 0.1). Results of ANOVAs run for the number of conserved positions for the whole genome (D). The vertical axis represents the proportion of conserved positions. There was a significant tissue main effect (p-values < 0.1) at the genome scale. Tissues are indicated as BM (bone marrow), LN (lymph node), and SP (spleen); infection status is indicated by sF (single infection with FIV) and dF (dual infection with FIV and PLV).
Additional file 2:
**All significant substitutions at individual site analysis.**

Additional file 3:
**Supplement Tables.**

Additional file 4: Figure S2.
**FIV Genome (9466 bp) Organization With Position Of The 4603 Bp Target Sequence Depicted As Solid Bar.** This sequence includes the genes *pol(ui)*: 4187--5239, *vif*: 5241--5996, *orfA*: 5997--6233, *env*: 6271--8789.
Additional file 5: Figure S3.
**Exponential-Normal Convolution Model.** Following the RMA approach, the overall mode serves as an estimate of the error mean μ, and the data on the left of such mode is used to estimate the error standard deviation σ. The signal rate α is estimated as in RMA-75, anchoring the 75^th^ percentile of an exponential distribution to the 75^th^ percentile of all frequencies minus the estimate of μ (i.e. the overall mode).
Additional file 6: Figure S4.
**Distribution Of The Asymmetry Index Across Sites.** The frequency of the asymmetry index (AI) across all sites and libraries (black) and at sites where the overall asymmetry [40] has a p-value of approximately zero (red).
