# Supplementary figures and images for: Error correction and statistical analyses for intra-host comparisons of feline immunodeficiency virus diversity from high-throughput sequencing data
Source: BMC Bioinformatics. 2015 Jun 30;16:202. doi: 10.1186/s12859-015-0607-z (PMC4486422; doi:10.1186/s12859-015-0607-z)

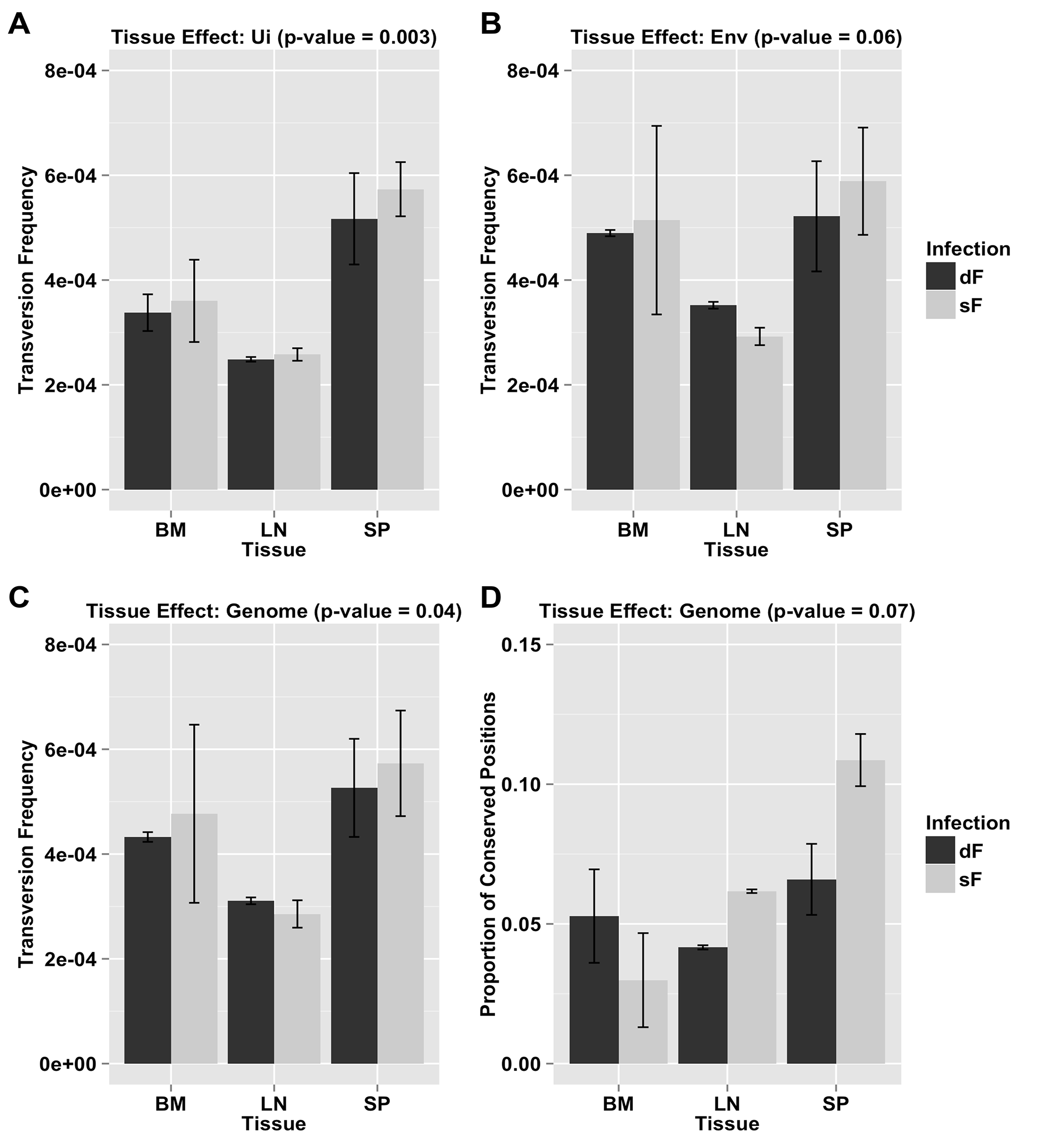

Supplement: Additional file 1: Figure S1. — Significant Tissue Main Effects For The Frequency Of Transversion And The Number Of Conserved Positions. Results of ANOVAs run for frequency of transversions (after logit transformations) in the FIV UI (A), and env (B) genes, and for the whole genome (C). The vertical axis represents transversion frequency. The dUTPase and integrase portion of the FIV pol (UI), envelope (env) genes, and the whole genome showed significant tissue main effects (p-values < 0.1). Results of ANOVAs run for the number of conserved positions for the whole genome (D). The vertical axis represents the proportion of conserved positions. There was a significant tissue main effect (p-values < 0.1) at the genome scale. Tissues are indicated as BM (bone marrow), LN (lymph node), and SP (spleen); infection status is indicated by sF (single infection with FIV) and dF (dual infection with FIV and PLV). [file 12859_2015_607_MOESM1_ESM.tiff]

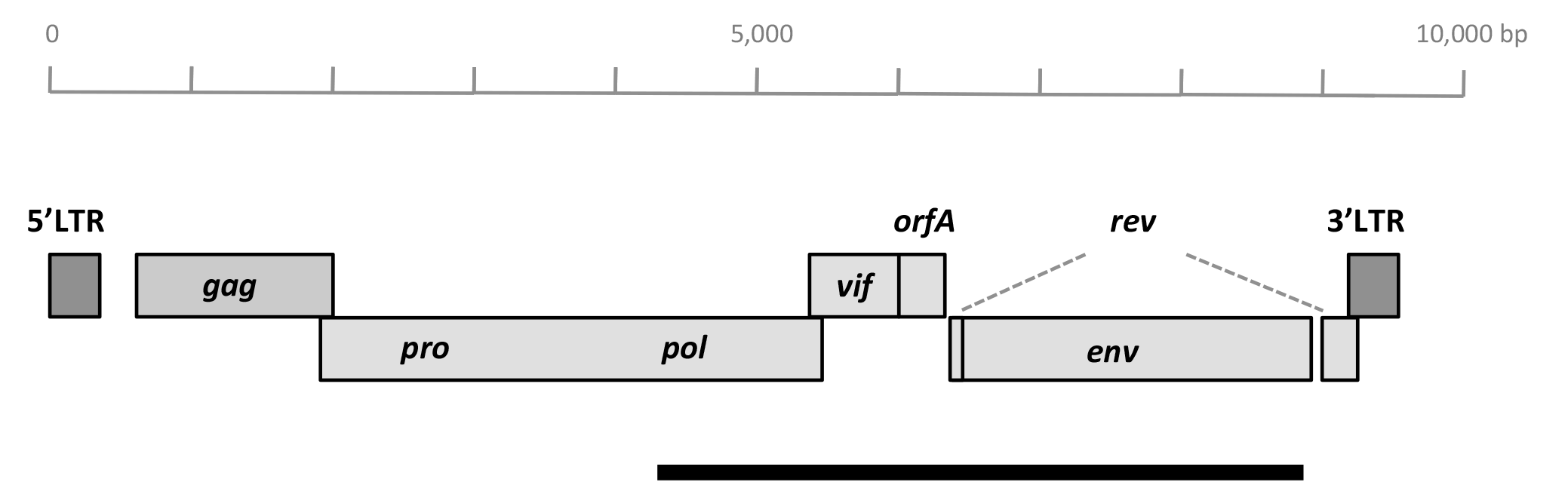

Supplement: Additional file 4: Figure S2. — FIV Genome (9466 bp) Organization With Position Of The 4603 Bp Target Sequence Depicted As Solid Bar. This sequence includes the genes pol(ui): 4187--5239, vif: 5241--5996, orfA: 5997--6233, env: 6271--8789. [file 12859_2015_607_MOESM4_ESM.tiff]

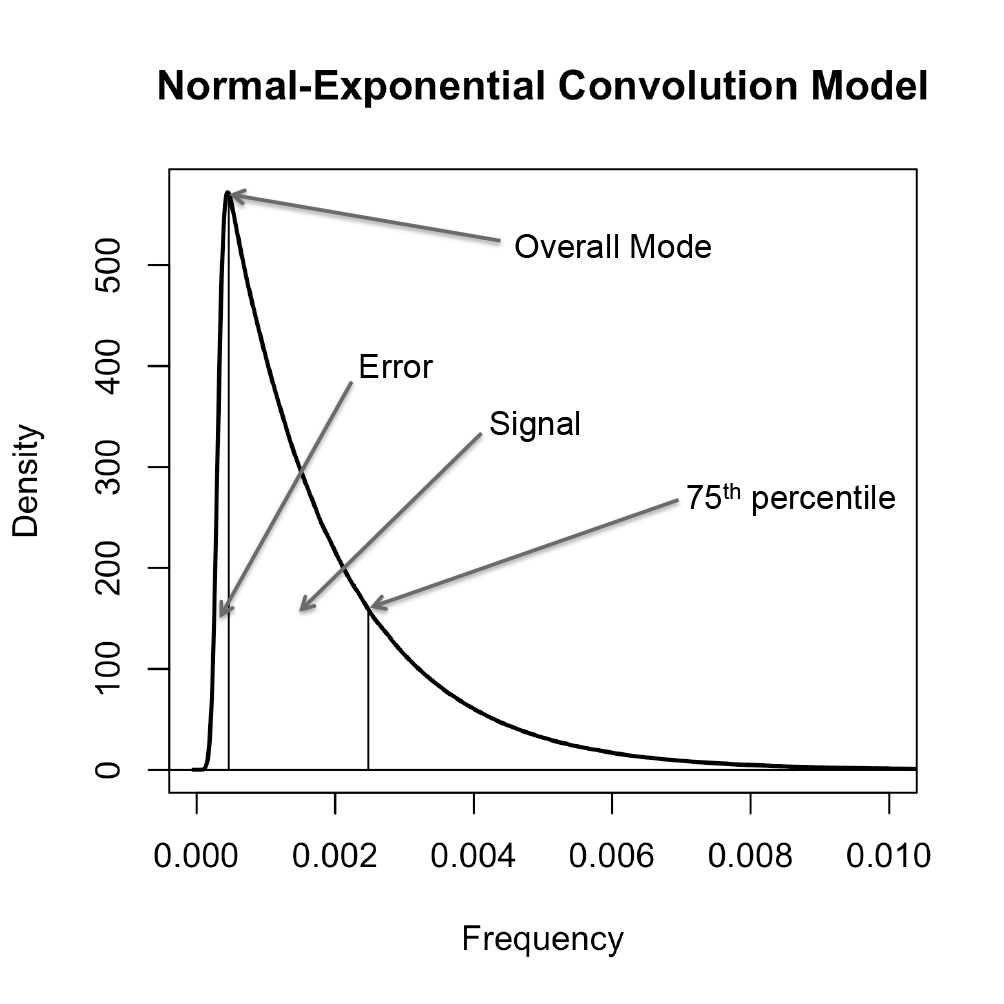

Supplement: Additional file 5: Figure S3. — Exponential-Normal Convolution Model. Following the RMA approach, the overall mode serves as an estimate of the error mean μ, and the data on the left of such mode is used to estimate the error standard deviation σ. The signal rate α is estimated as in RMA-75, anchoring the 75th percentile of an exponential distribution to the 75th percentile of all frequencies minus the estimate of μ (i.e. the overall mode). [file 12859_2015_607_MOESM5_ESM.tiff]

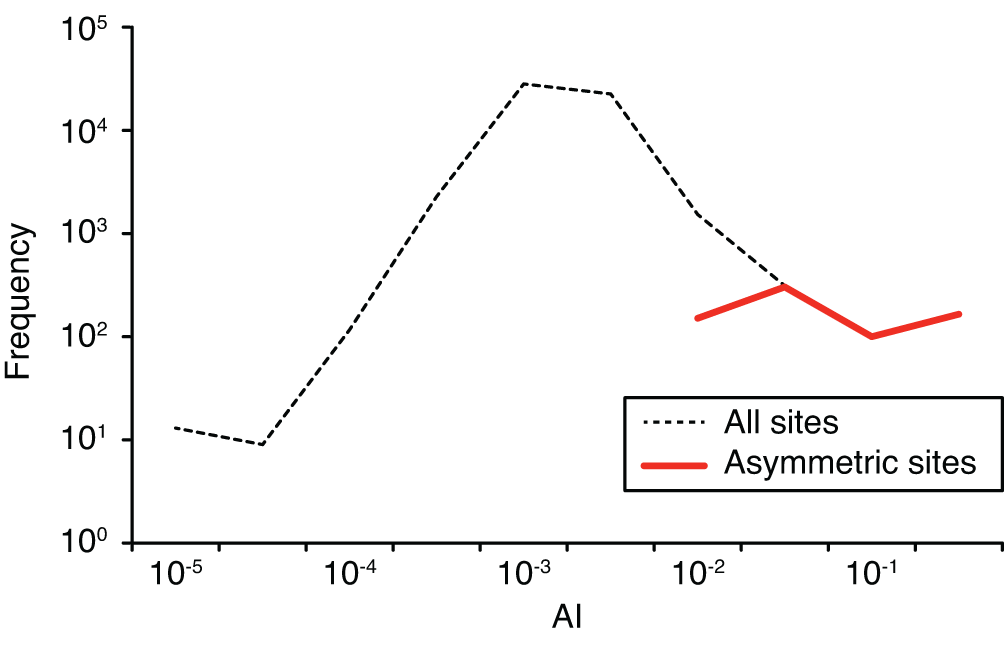

Supplement: Additional file 6: Figure S4. — Distribution Of The Asymmetry Index Across Sites. The frequency of the asymmetry index (AI) across all sites and libraries (black) and at sites where the overall asymmetry [40] has a p-value of approximately zero (red). [file 12859_2015_607_MOESM6_ESM.tiff]
