# Supplementary material for: Error correction and statistical analyses for intra-host comparisons of feline immunodeficiency virus diversity from high-throughput sequencing data
Source: BMC Bioinformatics. 2015 Jun 30;16:202. doi: 10.1186/s12859-015-0607-z (PMC4486422; doi:10.1186/s12859-015-0607-z)
Supplement: Additional file 3: — Supplement Tables. [file 12859_2015_607_MOESM3_ESM.docx]

**Supplementary Tables**

**Table S1 -** **Number of Minor Alleles Before and After Removal with Different Cut-offs, and ShoRHA Correction.**

| Library | No. Minor Alleles  (before correction) | No. Minor Alleles  (with 1% cutoff) | No. Minor Alleles  (with 0.05% cutoff) | No. Minor Alleles  (with 0.023% cutoff) | No. Minor Alleles  (with correction from ShoRAH) |
| --- | --- | --- | --- | --- | --- |
| 1 | 6253 | 96 | 5957 | 6253^a^ | 286 |
| 2 | 6526 | 75 | 5202 | 6526^a^ | 288 |
| 3 | 7911 | 83 | 5810 | 7666 | 534 |
| 4 | 7083 | 63 | 5150 | 6919 | 393 |
| 5 | 7314 | 123 | 4966 | 7092 | 405 |
| 6 | 8588 | 45 | 4980 | 6887 | 510 |
| 7 | 7839 | 51 | 5126 | 7280 | 405 |
| 8 | 8344 | 47 | 5241 | 7390 | 534 |
| 9 | 7581 | 55 | 5106 | 7469 | 395 |
| 10 | 10965 | 41 | 6806 | 8915 | 1565 |
| 11 | 7711 | 60 | 5203 | 7224 | 675 |
| 12 | 8744 | 32 | 5710 | 7964 | 711 |

^a^ No minor alleles frequencies in libraries 1 and 2 are smaller or equal to 0.023%.

**Table S2 - Significant Interaction and Tissue Effects with Different Error Correction Approaches**

| Level | Response | Effect | p-value  (0.05% cutoff) | p-value  (0.023% cutoff) | p-value  (0.023% cutoff + frequency correction) | p-value  (ShoRAH) |
| --- | --- | --- | --- | --- | --- | --- |
| *orfA* gene | Transition | Interaction | 0.04 | 0.04 | 0.04 | 0.25 |
| Ui gene | Conserved Sites | Interaction | 0.05 | 0.08 | 0.08 | 0.27 |
| *env* gene | Conserved Sites | Interaction | 0.42 | 0.1 | 0.1 | 0.59 |
| Ui gene | Transversion | Tissue | 0.002 | 0.01 | 0.003 | 0.002 |
| *env* gene | Transversion | Tissue | 0.07 | 0.09 | 0.06 | 0.20 |
| Genome | Transversion | Tissue | 0.05 | 0.07 | 0.04 | 0.17 |
| Genome | Conserved Sites | Tissue | 0.48 | 0.07 | 0.07 | 0.23 |

**Table S3 -** **Running Times for Error Correction Steps**

| Library | Convolution Model | ShoRAH |
| --- | --- | --- |
| 1 | 0.25s | 3h 58min |
| 2 | 0.26s | 8h 04min |
| 3 | 0.34s | 18h 17min |
| 4 | 0.31s | 66h 44min |
| 5 | 0.31s | 72h 15min |
| 6 | 0.38s | 152h 28min |
| 7 | 0.34s | 99h 00min |
| 8 | 0.37s | 102h 19min |
| 9 | 0.33s | 87h 59min |
| 10 | 0.62s | 80h 56min |
| 11 | 0.35s | 94h 02min |
| 12 | 0.40s | 29h 23min |

**Table S4 - FIV Proviral Load (copies per million cells)**

| Cat Number | Lymph Node | Spleen | Bone Marrow |
| --- | --- | --- | --- |
| 02 | 3266 | 2480 | 30961 |
| 99 | 1245 | 353 | 3120 |
| 06 | 5501 | 465 | 17600 |
| 97 | 7964 | 3147 | 18624 |
| 89 | 4115 | 543 | 14245 |
| 93 | 8087 | 668 | 11087 |
| 03 | 6911 | 1817 | 15019 |
| 05 | 2420 | 952 | 8906 |
